# Origins of Stochasticity and Burstiness in High-Dimensional Biochemical Networks

**DOI:** 10.1155/2009/362309

**Published:** 2008-05-14

**Authors:** Simon Rosenfeld

**Affiliations:** 1 Division of Cancer Prevention (DCP), National Cancer Institute, EPN 3108, 6130 Executive Blvd, Bethesda, MO 20892, USA

## Abstract

Two major approaches are known in the field of stochastic dynamics of intracellular biochemical networks. The first one places the focus of attention on the fact that many biochemical constituents vitally important for the network functionality may be present only in small quantities within the cell, and therefore the regulatory process is essentially discrete and prone to relatively big fluctuations. The second approach treats the regulatory process as essentially continuous. Complex pseudostochastic behavior in such processes may occur due to multistability and oscillatory motions within limit cycles. In this paper we outline the third scenario of stochasticity in the regulatory process. This scenario is only conceivable in high-dimensional highly nonlinear systems. In particular, we show that burstiness, a well-known phenomenon in the biology of gene expression, is a natural consequence of high dimensionality coupled with high nonlinearity. In mathematical terms, burstiness is associated with heavy-tailed probability distributions of stochastic processes describing the dynamics of the system. We demonstrate how the "shot" noise originates from purely deterministic behavior of the underlying dynamical system. We conclude that the limiting stochastic process may be accurately approximated by the "heavy-tailed" generalized Pareto process which is a direct mathematical expression of burstiness.

## 1. Introduction

High-dimensional biochemical networks are the integral parts of intracellular organization. The most prominent roles in this organization belong to genetic regulatory networks [1] and protein interaction networks [2]. Also, there are numerous other subsystems, such as metabolic [3] and glycomic networks [4], to name just a few. All these networks have several important features in common. First, they are highly diverse, that is, contain numerous (up to tens of thousands) different *types* of molecules. Second, their dynamics is constrained by a highly structured, densely tangled intracellular environment. Third, their constituents are predominantly macromolecules interacting in accordance with the laws of thermodynamics and chemical kinetics. Fourth, all these networks may be called "unsupervised" in the sense that they do not have an overlying regulatory structure of a nonbiochemical nature. Although the term "regulation" is frequently used in the description of cellular processes, its actual meaning is different from that in the systems control theory. In this theory, the regulatory signal produced by the controller and the way it directs the system are of a different physical nature than the functions of the system under control. In contrast, the intra- and intercellular regulations are of a biochemical nature themselves (e.g., protein signal transduction [5]); therefore, the subdivision of a system on the regulator and the subsystem-to-be-regulated is largely nominal. In order to be a stabilizing force, a biochemical "controller" should first be stable itself. Logically, such a subdivision serves as a way of compartmentalizing a big biochemical system into relatively independent parts for the simplification of analysis. However, in biology this compartmentalization is rarely unambiguous, and it is never known for sure what regulates what. An indiscriminate usage of the concepts and terminology borrowed from the systems control theory obscures the fundamental fact that intracellular functionality is nothing else than a vast system of interconnecting biochemical reactions between billions of molecules belonging to tens of thousands of molecular species. Therefore, studying general properties of such large biochemical systems is of primary importance for understanding functionality of the cell.

In this work, the focus of attention is placed on the dynamical stability of biochemical networks. First, we show that stringent requirements of dynamical stability have very little chance to be satisfied in the biochemical networks of sufficiently high order. The problem we encounter here is essentially of the same nature as in now classic work by May [6] where the famous question "will a large complex system be stable?" has been discussed in ecological context. Second, we show that a dynamically unstable system does not necessarily end its existence through explosion or implosion, as prescribed by simple linear considerations. It is possible that such a system would reside in a dynamic state similar to a stationary or slowly evolving stochastic process. Third, we conjecture that the motion in a high-dimensional system of strongly interacting units inevitably includes a pattern of "burstiness," that is, sporadic changes of the state variables in either positive or negative directions.

In biology, burstiness is an experimentally observed phenomenon [7–10], and a variety of theoretical approaches have been developed to understand its origins. Two of them have been especially successful in explanation of the phenomenon of burstiness. In the first one, the focus of attention is placed on the fact that many biochemical constituents vitally important for the network functionality may be present only in small quantities within the cell, and therefore, the regulatory process is essentially discrete and prone to relatively big fluctuations [11, 12]. The second approach treats the regulatory process as essentially continuous. Complex pseudostochastic behavior in such processes may occur due to multistability and oscillatory motions within limit cycles. An extensive summary of this line of theoretical works may be found in [13, 14]. There are numerous other approaches of various levels of mathematical sophistication and adherence to biological realities that attempt to explain the phenomenon of burstiness. It is far beyond the goals of this work to provide a detailed review. Recently published papers [15, 16] are good sources of more comprehensive information. In summary, the origins of stochasticity are so diverse that none of the existing theories may claim to be exhaustive. Each set of unmodeled realities in the system being modeled manifests itself as an additional stochastic force or noise. Stochasticity occurs at all levels of intracellular organization, from a single biomolecule, through the middle-size regulatory units, all the way up to tremendously large and complex systems such as GRN; each of these contexts requires a special tool for mathematical conceptualization.

The goal of this paper is to present a novel scenario of bursting, in addition to the existing ones. Unlike the approaches mentioned above, the mechanism we consider does not require any special conditions for its realization. Rather, it is seen as a ubiquitous property of any high-dimensional highly nonlinear dynamical system, including biochemical networks. The mechanism of stochastic behavior proposed here allows for some experimentally verifiable predictions regarding global parameters characterizing the system.

Interrelations between the stochastic and deterministic descriptions of multidimensional nonlinear systems, in general, and the systems of chemical reactions, in particular, have been given considerable attention in the literature [17–20]. It often happens, however, that an approach, being multidimensional theoretically, stumbles upon insurmountable mathematical difficulties in applications. As a result, there is often a big gap between the sophistication and generality of a theory, on one hand, and simplicity and particularity of the applications, on the other. A big promise in studying really large systems is seen in computational models, the ones that are capable of dealing with dozens [21] or even hundreds [22–24] of simultaneous biochemical constituents. These models, however, are necessarily linked to particular systems with all the specifics of their functionality and experimentally available parameterization. Due to these narrowly focused designs, computational models are rarely generalizable to other systems with different parameterizations; hence, common features of all such systems are not readily detectable. In addition, so far even big computational models are still too small to be able to capture global properties and patterns of behavior of really big biochemical networks, such as GRN.

The novelty of our approach consists of direct utilization of the property of the system to be "asymptotically diverse"; the bigger the system, the better the approximation we utilize is working. In the biochemical context, the term "asymptotically diverse" does not simply mean that the number of molecules in the system is very large; more importantly, it means that the number of individual molecular *species* is also very large, and that each of these species requires an individual equation for the description of its dynamics. In this paper, our goal is not in providing a detailed mathematical analysis of any particular biochemical system; rather it is to envision some important global properties and patterns of behavior inherent in the entire class of such systems. The novel message we intend to convey is that burstiness is a fundamental and ubiquitous property of asymptotically diverse nonlinear systems (ADNS). Of course, it would be an oversimplification to ascribe the burstiness in gene expression solely to the property of burstiness of ADNS. Nevertheless, there is little doubt that many subsystems in intracellular dynamics indeed may be seen as ADNS [25], and as such they may share with them, at least in part, the property of burstiness.

The problem of transition from deterministic to chaotic dynamics in multidimensional systems has long history in physics and mathematics, and a number of powerful techniques have been proposed to solve it [26–29]. It is rarely, however, the case that full strength of these techniques can be actually applied to real systems; far reaching simplifications are unavoidable. Preliminary qualitative exploration supported by partial theoretical modeling and simulation is a necessary step towards developing a theoretically sound yet mathematically tractable approximation. This paper, together with [30], is intended to provide such an exploration.

## 2. Nonlinear Model and State of Equilibrium

A natural basis for the description of chemical kinetics in a multidimensional network is the power-law formalism, also known under the name S-systems [24, 31–33]. Being algebraically similar to the law of mass action (LMA), S-systems proved to be an indispensable tool in the analysis of complex biochemical systems and metabolic pathways [34]. A useful property of S-systems is that S-functions are the "universal approximators," that is, have the capability of representing a wide range of nonlinear functions under mild restrictions on their regularity and differentiability. S-functions are found to be helpful in the analysis of genome-wide data, including those derived from microarray experiments [35, 36]. However, the most important fact in the context of this work is that in the vicinity of equilibrium *any* nonlinear dynamical system may be represented as an S-system [37]. Unlike mere linearization, which replaces a nonlinear system by the topologically isomorphic linear one, the S-approximation still retains essential traits of nonlinearity but often is much easier to analyze.

In the S-system formalism, equations of chemical kinetics may be recast in the following form:(1)

where  are the rates of production and degradation, and  are the stoichiometric coefficients in the direct and inverse reactions, respectively. Depending on the nature and complexity of the system under investigation, the quantities  may represent various biochemical constituents participating in the process, including individual molecules or their aggregates. There is no unique way of representing the biochemical machinery in mathematical form: depending on the level of structural "granularity" and temporal resolution, the same process may be seen either as an individual chemical reaction or as a complex system of reactions. For example, on a certain level of abstraction, the process of transcription may be seen as an individual biochemical reaction between RNA polymerase and DNA molecule, whereas a more detailed view reveals a complex "dance" involving hundreds of elemental steps, each representing a separate chemical reaction [38, 39]. Formally, the system of S-equations (1) is analogous to the equations of chemical kinetics in which each constituent is generated by only one direct and only one reverse reaction. Reality of large biochemical systems is, of course, far more complex. In particular, there may be several competitive reactions producing and degrading the same constituents but following different intermediate pathways. For these cases, a more appropriate form of the equations would be(2)

known as the law of generalized mass action (GMA). Here  are the numbers of concurrent reactions of production and degradation,  are the matrices of rates, and  are the tensors of stoichiometric coefficients. However, in principle, this more complex system is reducible to form (1) by appropriate redefinition of chemical constituents [40]. Even more important is the fact that *any* nonlinear dynamical system, after a certain chain of transformations, may be represented in the form (1); for this reason this form is sometimes called "a canonical nonlinear form" (see [32], and also [41, 42]). At last, as it has been recently shown in [37], in the vicinity of equilibrium, a wide class of nonlinear systems is topologically isomorphic to the canonical S-system (Appendix A).

Simple algebra allows for transformation of (1) to a more universal and analytically tractable form: (3)

where  is the rescaled time, , and  is the set of constants characterizing constituent-specific rates of chemical transformations (see [30, 43] and Appendix B for definitions and technical details; for simplicity of notation,  is further replaced by ).

It is easy to see now that the fixed point of (3) is located in the origin of coordinates and that the Jacobian matrix in its vicinity is simply (4)

No simplifications have been made for the derivation of (3). This means that these equations are quite general and may be always derived for any given sets of rates and stoichiometric coefficients.

## 3. Structure of the Solution in the Vicinity of Equilibrium

Equations in (3) may be simultaneously viewed as renormalized equations of chemical kinetics derived from and governed by the laws of nonequilibrium thermodynamics, and also as the equations of an abstract dynamical system, whether originating in chemistry or not. There is a fundamental difference between the dynamic equilibrium resulting from the conditions , and the thermodynamic equilibrium expressed in the LMA in chemical kinetics [44]. The latter assumes, in addition to the fact that the fixed point is the equilibrium point, existence of the detailed balance, that is, full compensation of each chemical reaction by the reverse one. For an arbitrary dynamical system, there are no first principles that would impose any limitations on the structure of the Jacobian matrix, , in the vicinity of the fixed point. This means, in turn, that  is just a matrix of general form having the eigenvalues with both positive and negative real parts. Consequently, there are no reasons to assume that the macroscopic law of motion for such systems, that is, , is stable. Although the assumption of stability is frequently introduced in the context of genetic regulation, in fact, it refers to a highly specific condition which is hardly possible in an *unsupervised* multidimensional system with many thousands of independent governing parameters.

In this context, it is useful to recall some fundamental results pertaining to stability of nonlinear systems. According to the theorem by Lyapunov, the matrix  is stable if and only if the equation  has a solution, , and this solution is a *positive definite* matrix [45]. Matrix , if exists, is a complicated function of all the stoichiometric coefficients and kinetic rates characterizing the network. Thus, the Lyapunov criterion would impose a set of very stringent constraints of high algebraic order on the structure of dynamically stable biochemical networks. Another classical approach to stability consists of the application of the Routh-Hurwitz criterion [45]. In this approach, one first calculates the characteristic polynomial of the Jacobian matrix, and then builds the sequence of the so-called Hurwitz determinants from its coefficients. The system is stable if and only if *all* the Hurwitz determinants are positive. Again, the Routh-Hurwitz criterion imposes a set of very complex constraints on the global structure of a biochemical network. As argued above, apart from the principle of detailed balance (PDB), there are no other first principles and/or general laws governing stability of biochemical systems, and neither the Lyapunov nor the Routh-Hurwitz criteria are the corollaries of PDB. As shown in [43], the Jacobian matrix of an arbitrary biochemical system may have comparable numbers of eigenvalues with negative and positive real parts. This property holds under widely varying assumptions regarding kinetic rates and stoichiometric coefficients. Therefore, generally, high-dimensional biochemical networks which are not purposefully designed and/or dynamically stabilized (e.g., as in the reactors for biochemical synthesis [46]) are reasonably presumed to be unstable. Considerable efforts have been undertaken to infer global properties of large biochemical networks far from thermodynamical equilibrium from the first principles; many notable approaches have been developed up to date. Among them are the chemical reaction network theory [47], stoichiometric network theory [48], thermodynamically feasible models [49], imposing constraints of microscopic reversibility [50], minimal reaction scheme [51], to name just a few. However, in the majority of these approaches, stability, either dynamical or stochastic, is presumed a priori and serves as a starting point for further considerations. These theories neither question the existence of such stability nor explain why a big biochemical network *should* necessarily be stable.

## 4. Stochastic Cooperativity and Probabilistic Structure of Burstiness

**Figure 1:**
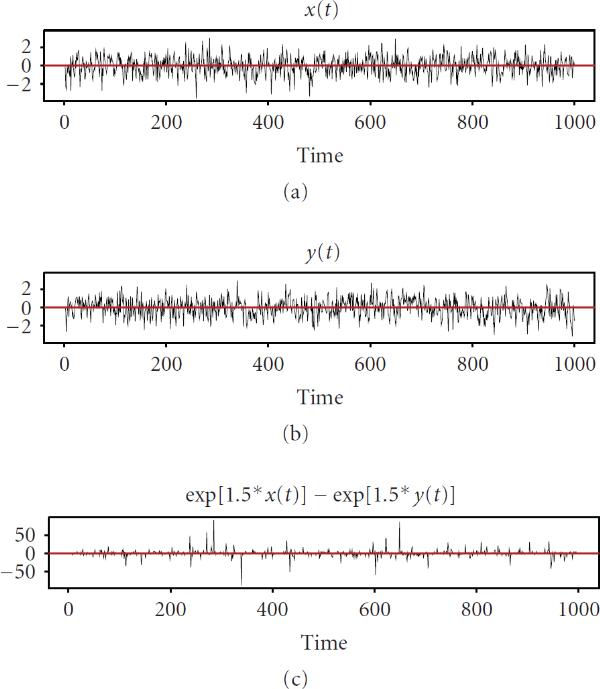
**Illustration of the notion of burstiness**.

**Figure 2:**
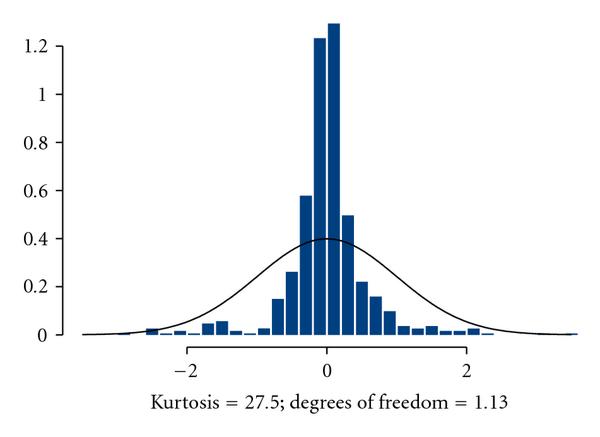
**Histogram of the process depicted in Figure 1.** The distribution is close to the Student's *t* with number of degrees of freedom 1.13. This is an indicator of "heavy tails." Solid line belongs to the standard normal distribution, .

The term cooperativity is widely used in biology for describing multistep joint actions of biomolecular constituents to produce a singular step in intracellular regulation [52, 53]. In intracellular regulatory dynamics, the term cooperativity reflects the fact that an individual act of gene expression is not possible until all the gene-specific coactivators are accumulated in the quantities sufficient for triggering the transcription machinery. In ODE terms, this means that  in (3) may noticeably deviate from zero only when the majority of arguments in  and  come to "cooperation" by simultaneously reaching vicinities of their respective maxima. This notion is illustrated by the following simple example. Let us assume that  and  are random, not necessarily Gaussian, processes with identical statistical characteristics, and consider the behavior of the process, . The pattern of this behavior is seen in Figure 1 whereby  fluctuates in the vicinity of zero most of the time, thus making no contribution to the variations of . However, sometimes  makes large excursions in either direction causing fast sporadic changes in . As shown in Figure 2, the distribution of  is approximately symmetric. This means that positive excursions are generally balanced by negative ones. This observation helps us to understand how it happens that an inherently unstable system nevertheless behaves decently and does not explode or implode as prescribed by its linear instability. In simplified terms, the reason is that sporadic deviations of concentrations in positive directions are followed, sooner or later, by the balancing responses in degradation, thus maintaining approximate equilibrium.

**Figure 3:**
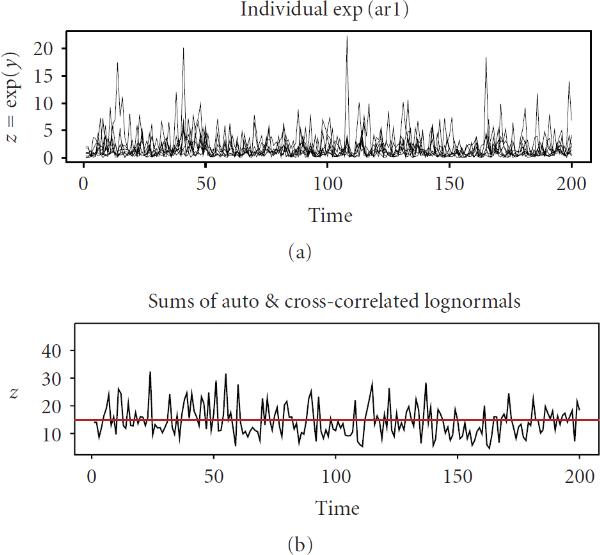
**Convergence of the sums of lognormal processes (a) to approximate normality (b)**.

**Figure 4:**
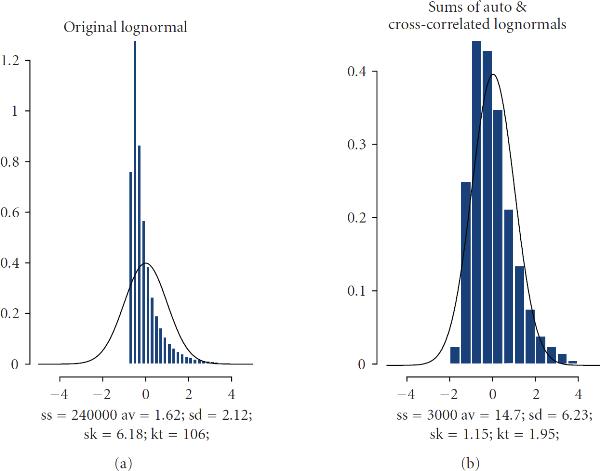
**Illustration of convergence to normality.** The histograms belong to processes shown in Figure 3. (a) Lognormal processes (skeweness 6.2, kurtosis 106). (b) Distribution of sums of 80 lognormals (skeweness 1.2, kurtosis 2). In both cases, solid lines belong to standard normal.

In order to envision stochastic structure of the solution to (3), we make use of three fundamental results from the theory of stochastic processes, namely, (i) central limit theorem (CLT) under the strong mixing conditions (SMC) [54]; (ii) asymptotic distribution of level-crossings by stationary stochastic processes [55], and (iii) probabilistic structure of heavy-tailed (also known as bursting) processes [56]. We first notice that the arguments of  in (3) are combined into two linear forms, (5)

in which only  terms are nonzeros, where  is the typical number of transcriptional coactivators facilitating gene expression; as mentioned above, this number may be of order from several dozens to hundreds. Generally, these collections of transcription factors are gene-specific, and there is no explicit correlation between transcription rates and transcription stoichiometry. According to the CLT under the SMC, the sums of weakly dependent random variables are asymptotically normal. Validity of the SMC, as applied to  and , is easy to demonstrate by simulation. Importantly, the sums (5) are asymptotically normal even when the processes  are nonGaussian. Figures 3 and 4 provide an illustration of convergence to normality. In this example, individual time series  are selected drastically nonnormal, namely lognormal, and average cross-correlation between  is selected on the level 0.15. Nevertheless, summation of only 80 series, , results in the stochastic processes,  and  which are fairly close to Gaussian. Thus, we conclude that  and  are approximately Gaussian (see [30] for more detail). Therefore, the processes  and  are lognormally distributed; their expectations and variances are, respectively, (6)

where dot stands for  or . The correlation coefficient between two exponentials is (7)

The right-hand side in (3) is the difference of two lognormal random variables. Exact probabilistic distribution of this difference is unknown. We have found by simulation that these distributions may be reasonably well approximated by the generalized Pareto distribution (GPD): (8)

**Figure 5:**
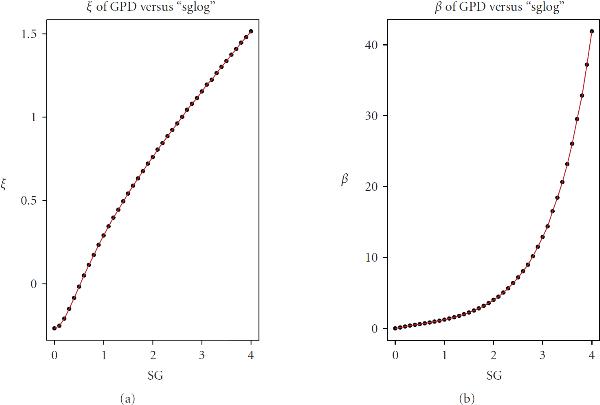
**Parameters of GPD expressed through the standard deviation,**. Dots are the parameters obtained by fitting the GPD to the simulated ; solid lines are the parameters obtained through the analytical approximations (10)-(11).

More specifically, the *tail* distributions of (9)

may be accurately represented through (8) with appropriately selected parameters  and . These dependencies are shown in Figure 5. Furthermore, very accurate analytical approximations are available for  and . It turns out that  is nearly linear: (10)

and  is nearly exponential: (11)

**Figure 6:**
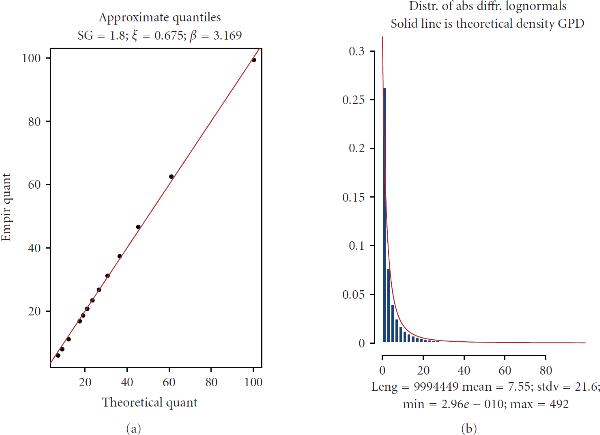
**Example of approximation of the difference of two lognormals by the GPD**. (a) QQ-plot of theoretical GPD versus empirical ; (b) empirical histogram of  versus theoretical GPD density.

Although the primary goal for these approximations is to accurately capture only the *tail* distributions of , nevertheless within the interval  approximations (10)-(11) are found to be quite satisfactory down to 0.1-quantile. Essentially, this means that GPD may serve as a very good representation for  as a whole, not just for the tails. Figure 6 shows an example of fitting the GPD to . The histogram in Figure 6(b) depicts empirical distribution of  resulting from the Monte Carlo simulation; a solid envelopeline belongs to the theoretical density of GPD with parameters  and  obtained from (10)-(11).

**Figure 7:**
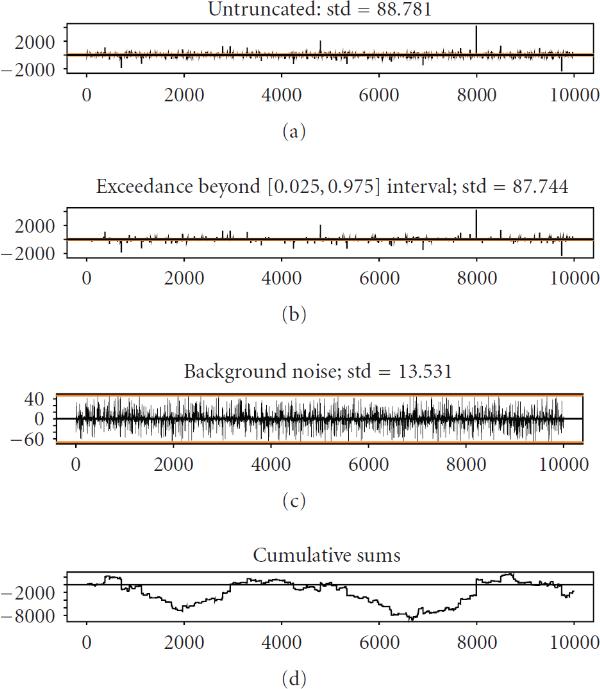
**(a) Process **. (b) Process of exceedances . (c)Residual noise, . (d) Trajectory of the random walk generated by . Note that the variance of residual noise, , is only 2.3% of total variance , despite the fact that exceedances, , occupy only 5% of the probability space.

The fact that  is representable through the heavy-tailed GPD is significant. As well known from the literature [56], stochastic processes with heavy-tailed distribution usually possess the property of burstiness. This property means that a substantial amount of spectral energy of such processes is contained in exceedances, that is, in the short sporadic pulses beyond the certain predefined bounds. Figure 7 illustrates this concept. Figure 7(a) depicts the stochastic process (12)

where  and  are standardized independent Gaussian processes. Figure 7(b) shows the process of exceedances, , defined as the part of  jumping outside the interval . Although  spends only 5% of all the available time outside this interval, its variance is overwhelmingly greater than that of difference,  (resp., 183 and 7698). On this basis, we may regard  as a small background noise which only slightly distorts the strong signal provided by . If we ignore this noise, then (12) acquires a familiar form of the Langevin equation (13)

where  is the matrix of random Pareto-distributed amplitudes and  is the set of random point processes coinciding with the events of bursting. Transition from (3) to (13) signifies replacement of purely deterministic dynamics by the *pseudostochastic* process similar to shot noise. We emphasize again that no assumptions have been made regarding *extrinsic* noise of any nature which may be present in a dynamical system and which is frequently used as a vehicle for introducing a stochastic element into the system's behavior [17, 57]. The point we make is that even in the absence of such an external source of stochasticity, a multidimensional system itself generates a very complex behavior which *for all practical purposes* may be regarded as a stochastic process. Formally, this type of stochasticity may be regarded as a case of chaotic dynamics, but it is fundamentally different from what is usually assumed under the terms chaos or chaotic maps in the literature. As known from the literature, chaotic behavior may appear even in a low-dimensional system with a very simple structure of nonlinearity, such as in the celebrated example of Lorenz attractor [58]. Usually in such systems, the bifurcations with transition to chaos appear under highly peculiar conditions expressed in a precise combination of the parameters governing the system. In this sense, chaos is not something typical of low-dimensional nonlinear systems, but rather is a rare and coincidental exclusion from the majority of smoothly behaving systems with a similar algebraic structure. On the contrary, in the model proposed in this work, stochasticity emerges under very general and quite natural conditions without any special requirements imposed on the governing parameters. In this sense, this kind of stochasticity may be regarded as a highly typical all-pervading pattern in the behavior of high-dimensional highly nonlinear dynamical systems.

**Figure 8:**
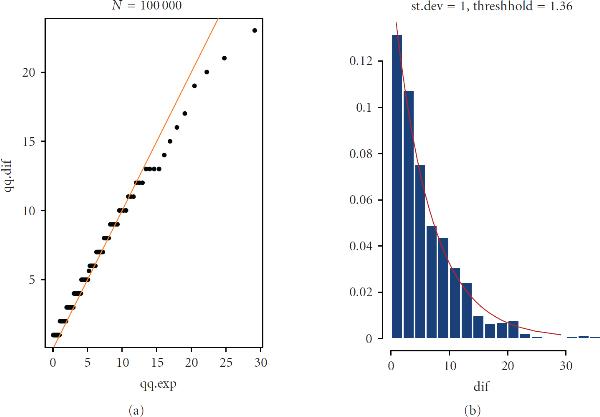
**Evidence that the exceedances form a Poisson process: waiting times are exponentially distributed.** The number of peaks predicted from asymptotic theory is 703; the number actually found in simulation is 695.

These heuristic considerations are supported by simulation. Temporal locations of pulses, , are those corresponding to local maxima of  and . We compare their probabilistic properties of their exceedances with those known from the theory of genuinely stochastic processes. It is a well-known result from the theory of level-crossing processes [55] that the sequence of such events in the interval  asymptotically, , converges to a Poisson process with the parameter (14)

where  is the threshold of excursion; and  and  are the correlation radius and variance of the generating Gaussian processes, respectively. On the basis of this asymptotic result, it may be reasonably assumed that for a finite, but sufficiently large , the sequences, , may also form a set of Poisson processes with appropriately selected parameters. Figure 8 shows an example of simulation where the threshold, , is not big at all, it is only slightly greater than the standard deviation, . The QQ-plot and histogram of waiting times, , clearly follow exponential distribution, which is an indication that the sequence  forms a Poisson process. It is also worth mentioning that in this simulation the number of peaks in the interval  predicted from the asymptotic theory, 703, is fairly close to the number of peaks actually found, 696. These two findings indicate that (14) is practically applicable under much milder conditions than .

## 5. Fokker-Plank Equation and Global Behavior

Having the Langevin equation (12) in place, we may now derive the corresponding Fokker-Plank equation (FPE). For this purpose, we compute increments, (15)

over the period of time, , encompassing many excursion events. Since , we have the following equation for the variances of increments.(16)

Denoting (17)

and using the standard Dirichlet technique, we find (18)

By definition, the diffusion coefficient is (19)

Since the correlation radius is much smaller than the interevent time, in the above integral  may be extended to . Therefore, (20)

Integrand in the expression (20), after some inessential simplifications, may be reduced to (21)

where  (see Appendix C for details). In (21),  are the autocorrelation functions of individual series . Applying the saddle point approximation to the integral (21), we come to the following expression for the diffusion coefficient (see Appendix D). (22)

where  denotes the network-wide variance of fluctuations and  is the network-wide square of relaxation time. Equation (22) reveals important details of multidimensional diffusion in the ADNS network. First, there is a common factor created by the entire network  which acts uniformly upon all the individual constituents. But also there are individual motilities characterized by the factors . Equation (22) means that all the constituent-specific concentrations, after being rescaled by their kinetic rates, , have the same diffusion coefficient, (23)

and therefore, satisfy the same univariate FPE. It is natural to assume that correlation times, , are of the same order of magnitude as the corresponding times of chemical relaxation, , because both introduce characteristic time scales into the individual chemical reactions. Therefore, the entire system may be stratified by only one set of parameters, the kinetic rates, .

Generally, the probabilistic state of a biochemical network may be characterized by joint distribution,  of all the chemical constituents which satisfies the multivariate FPE [59]. However, in light of the above simplifications, such a detailed description would be redundant. Instead, we introduce a collection of  identical univariate probability distributions, , where  is any of the , each satisfying *the same* FPE with the coefficient of diffusion (22). This self-similarity grossly simplifies analytical treatment of the problem. First, it means that variances, , are directly proportional to the squares of corresponding kinetic rates. Since , we conclude that , that is, in stationary fluctuations, the variances of logarithms of concentrations are proportional to the squares of kinetic rates. This is a testable property of all the large-scale biochemical networks; it may serve as a basis for experimental validation. Furthermore, since  is the only set of constituent-specific temporal scaling parameters in the network, it is natural to surmise that the times of correlation, , are directly proportional to the corresponding times of chemical relaxation, . This is another macroscopically observable property suitable for experimental validation.

Due to random partitioning and stochasticity of transcription initiation [60, 61], initial conditions for the system's evolution are considered as random. Starting with these initial conditions, the system is predominantly driven by the sequence of sporadic events of stochastic cooperativity. Although each event produces a noticeable momentary shift in the system's evolution, the multitude of such events makes its overall behavior quite smooth. This behavior is illustrated in Figure 7(d). Smoothness of the trajectories, in practical sense, may be regarded as macroscopic stability, whereas the deviations from these smooth trajectories may be seen as "noise."

As a side note, it is worth mentioning that in this paper, the Pareto representation of exceedances has been derived from the assumption that  and  are approximately Gaussian processes, and, therefore,  and  are approximately lognormally distributed. We have justified this closeness to normality of  and  by the CLT. This assumption, however, only served to simplify the analysis; it may be substantially relaxed at the expense of increased complexity of calculations. Conceptually, all the major ideas leading to the notion of stochastic cooperativity would stay in place even without transition to asymptotic normality. Let us assume again, as we did in the examples in Figures 3-4, that , where  are lognormal processes. This time, however, it is not assumed that the number of nonzero elements in these sums is sufficiently large to equate the distributions of sums to their asymptotic limits. This would reflect the situation when the number of transcription factors in GRN is comparatively small. Generally, exact analytical expressions for the distributions of sums of lognormals are unknown, but there is a consensus in the literature that such sums themselves may be accurately modeled as lognormally-distributed [62]. We have performed a simulation for studying the probabilistic structure of the exceedances with lognormal . It is rather remarkable that the GPD turns out to be a good approximation in this drastically nonnormal case as well; the only reservation should be made that simple parameterization (10)-(11) is no longer valid and should be replaced by a more complex one.

Summarizing all these findings, we conclude that inherent dynamical instability of the system considered as deterministic directly translates into heavy-tailness and burstiness in stochastic description. Sequence of events of stochastic cooperativity serves as a link between deterministic and stochastic paradigms.

## 6. Summary

We have outlined the mechanism by which a multidimensional autonomous nonlinear system, despite being dynamically unstable, nevertheless may be stationary, that is, may reside in a state of stochastic fluctuations obeying the probabilistic laws of random walk. Importantly, in this mechanism, the transition from the deterministic to probabilistic laws of motion does not require any assumptions regarding the presence of extraneous random noise; stochastic-like behavior is produced by the system itself. An important role in forming this type of fluctuative motion belongs to inherent burstiness of the system associated with the events of stochastic cooperativity. Unlike the classical Langevin approach, macroscopic laws of motion of the system are not required to be dynamically stable.

In this work, we have selected the S-systems to be an example of a nonlinear system. Three motivations justified this selection. First, the S-systems are structured after the equations of chemical kinetics, thus being a natural tool for description of high-dimensional biochemical networks. Second, many other nonlinear systems may be represented through the S-systems in the vicinity of fixed point. Third, despite generality, the S-systems have an advantage of being analytically tractable. However, many results regarding stochastic cooperativity and burstiness may be readily extended to other multidimensional nonlinear systems. In such a system, short pulses during the events of stochastic cooperativity may be described in terms of "shot" noise with subsequent derivation of the Fokker-Plank equation. As proposed in this paper, it is possible to indicate some general experimentally verifiable predictions regarding the behavior of this type of system, such as distribution of intensities of fluctuations and distribution of temporal autocorrelations among individual units of the system.

## Appendices

### A. Replacement of an Arbitrary Nonlinear Dynamics by the S-Dynamics

In this section, we follow the methodology outlined in [37] adapting the formulae and notation to the specific goals of this work. We consider the nonlinear system (A.1)

where  and  are monotonic functions, and  and  are the matrices with positive elements. We first select an *arbitrary* point  and expand  in the Taylor series in its vicinity (A.2)

where (A.3)

We denote (A.4)(A.5)

With definitions (A.5), (A.4) may be rewritten as (A.6)

thus bringing (A.1) to the standard form of S-system (A.7)

with the parameters dependent on .

The "tangential" system (A.7) has a unique fixed point, . To find it, we require that (A.8)

Denoting temporarily (A.9)

we find the equilibrium point conditional on (A.10)

We introduce the map, , and rewrite (A.10) as 

We may now select the point  as a new starting point, and deduce (A.11)

Tournier [37] proved that the point  which is the fixed point of , that is, , is also the fixed point of the (A.1), that is, , and that the sequence  converges to this point when . Therefore, we may conclude that in the vicinity of the fixed point, whether stable or unstable, general equations (A.1) may be rewritten in the form (A.12)

Formally, these equations may be seen as a system of equation of chemical kinetics with  and  being the rates,  and  being stoichiometric coefficients, and  being chemical constituents. It is not out of place to mention again, that since  and  are arbitrary vector functions, then there is no special symmetry in the Jacobian matrix of the system in the vicinity of fixed point. Therefore, there is no reason to expect that its eigenvalues have only negative real parts, that is, that the fixed point is stable.

### B. Derivation of (3)

Following the standard procedures in nonlinear dynamics [63], we first search for the state of dynamical equilibrium, , commonly referred to as a fixed point, that is, the point where all the time derivatives turn to zero, and which therefore satisfy (B.1)

Taking logarithms of both sides and solving the linear equations, we obtain the vector of solutions: (B.2)

Note that stoichiometric coefficients  and  cannot be identical *in all* the direct and inverse reactions simultaneously, therefore, the matrix  is always invertible.

It is convenient to introduce relative quantities,  and then, after denoting (B.3)

we obtain the equations (B.4)

where (B.5)

Since we are interested only in positive solutions, we replace  and obtain (B.6)

where , and .

We note further that (B.7)

and because , we find that , therefore, (B.8)

After introducing a more appropriate time scale , where , we rewrite (B.8) as (B.9)

where  with an important property that . It is easy to see that now the fixed point is located in the origin of coordinates and that the Jacobian matrix in the vicinity of this point is (B.10)

### C. Derivation of the Autocorrelation Function

By definition (C.1)

We have further, (C.2)

Similarly, (C.3)

At last, (C.4)

Putting everything together, (C.5)

The terms  are small compared to , first, because , and second, because the double sums here are of the same order of magnitude as the sums of variances. We, therefore, reduce (C.5) to (C.6)

### D. Derivation of the Diffusion Coefficient Using the Saddle Point Approximation

Let  be a decreasing function of  such that: . Then, (D.1)

Denoting , we find that  is a good representation of the integrand in (D.1) both in the vicinity of zero and at infinity. Therefore, (D.2)

Introducing , and , we obtain (D.3)

Denoting , we get  Therefore, (D.4)

Introducing the parameters (D.5)

we finally obtain (D.6)
